# The impact of COVID-19 on the mental and sexual health of patients with infertility: a prospective before-and-after study

**DOI:** 10.1186/s12958-023-01174-7

**Published:** 2024-01-02

**Authors:** Jing Qi, Meng Sun, Xingchen Yue, Xintong Hong, Meng Dong, Jichun Tan

**Affiliations:** 1 Center of Reproductive Medicine, Shengjing Hospital of China Medical University, No. 39 Huaxiang Street, Shenyang, China; 2 Department of Obstetrics and Gynecology, Shengjing Hospital of China Medical University, Shenyang, China; 3 NHC Key Laboratory of Advanced Reproductive Medicine and Fertility (China Medical University), National Health Commission, Shenyang, China; 4 Key Laboratory of Reproductive Dysfunction Diseases and Fertility Remodelling of Liaoning Province, Shenyang, China; 5 Northeast Yucai Foreign Language School, Shenyang, China

**Keywords:** COVID-19, Infertility, Sexual health, Mental health, Before-and-after study, Full-release

## Abstract

**Background:**

The coronavirus disease (COVID-19) pandemic has seriously impacted the mental and sexual health of the general population. Patients dealing with infertility constitute a unique subset within society, susceptible to heightened sensitivity amid pressures and crises. However, to the best of our knowledge, the impact of the different stages of the COVID-19 pandemic on the mental and sexual health of patients with infertility has not been investigated. Therefore, this study aimed to investigate the mental and sexual health of patients with infertility during different stages of the COVID-19 pandemic (during the lockdown, when controls were fully liberalized, and during the post-pandemic era).

**Methods:**

This prospective before-and-after study was conducted between April and May 2022 (during the lockdown), December and January 2023 (when controls were fully liberalized), and May and August 2023 (during the post-pandemic era). This study explored the sexual and mental health of women with infertility during the three stages of the COVID-19 pandemic using standardized mental health and sexual function questionnaires. The Chi-square test was used to compare categorical data, and the ANOVA test was used to compare numerical data.

**Results:**

Patients had the highest 7-item Generalized Anxiety Disorder Scale (GAD-7) and 9-item Patient Health Questionnaire (PHQ-9) scores and the highest rates of anxiety and depression during the immediate full-release phase. During the complete liberalization phase, patients had the lowest Female Sexual Function Index (FSFI) scores and the highest incidence of sexual dysfunction.

**Conclusion:**

This study is the first one to report the repercussions of COVID-19 on the mental and sexual well-being of individuals experiencing infertility across various phases of the pandemic. Upon the complete lifting of control measures, close to 99% of participants exhibited varying degrees of anxiety and depression. Our research underscores that individuals with infertility faced elevated levels of anxiety, depression, and sexual dysfunction during the phase of full liberalization of COVID-19 control measures, in stark contrast to the periods of lockdown and the post-pandemic era.

**Supplementary Information:**

The online version contains supplementary material available at 10.1186/s12958-023-01174-7.

## Introduction

The onset of the COVID-19 pandemic worldwide in 2020 brought unprecedented social and economic impacts, which have also had a substantial impact on the health of the population. Social lockdowns, quarantines, school and business closures, job losses, reduced economic activity, and various policies were implemented by governments to try to contain the coronavirus pandemic, which could and can seriously affect the mental health of the population [1]. Data from the Global Burden of Disease Study 2019 (GBD 2019) showed that the two most disabling mental disorders were depression and anxiety, both of which belonged to the top 25 leading causes of disease burden worldwide in 2019 [2]. In addition, healthcare professionals have focused on the impact of COVID-19 on sexual health [3]. The negative impact of COVID-19 on mental and sexual health may be altered by changes in measures related to disease control, such as policy development to prevent and control disease progression, leading to persistent fear and panic about the virus [4].

Infertility, recognized as a life crisis, profoundly impacts individuals globally [5]. The emotional toll of infertility is considerable, often giving rise to heightened levels of anxiety and stress among patients [6]. Patients dealing with infertility constitute a unique subset within society, susceptible to heightened sensitivity amid pressures and crises. The confluence of infertility and the unique stressors posed by the pandemic underscores the necessity for a comprehensive understanding of the mental health implications for this particular group [7]. Our investigation into the mental health conditions of individuals grappling with infertility predates the onset of the COVID-19 pandemic. A comprehensive review of existing literature revealed a pronounced correlation between infertility and heightened mental health challenges, surpassing those experienced by women with typical fertility [8]. Remarkably, 25–60% of individuals contending with infertility were found to grapple with mental health issues, notably anxiety and depression [8]. In a 2015 study conducted in the United States, 39% of 74 participants dealing with infertility were reported to be contending with depression [9]. Another study encompassing 352 women grappling with infertility unveiled that 56% exhibited overt depressive symptoms, while a staggering 76% manifested significant anxiety symptoms [6]. It is essential to acknowledge that the incidence of psychological problems can vary due to the diverse assessment scales employed across studies and differences in ethnic backgrounds. Nonetheless, a consistent pattern emerges, indicating that individuals facing infertility confront substantial mental health challenges.

COVID-19 has seriously impacted the mental and sexual health of the general population [10–12]. The challenges become even more pronounced amid the backdrop of the COVID-19 pandemic, where mental health concerns are exacerbated by factors like health anxieties, social distancing mandates, and pervasive life uncertainty [13]. Amidst the COVID-19 lockdown, a comprehensive set of measures was implemented, encompassing restrictions on the movement of individuals, home isolation protocols, closure of schools and businesses, daily COVID-19 throat swab testing, and other stringent measures. On December 7, 2022, China fully liberalized its epidemic control measures, which was followed by a “pandemic” of new crown infections and an increasing number of positive test results for COVID-19. In the face of the sudden and comprehensive liberalization of control, the population was infected on a large scale, and significant social phenomena such as “difficulty in seeking medical treatment” and “difficulty in buying medicine” appeared.

Although the impact of the COVID-19 pandemic on the mental and sexual health of populations has been reported, the impact of the different stages (during the lockdown, when controls were fully liberalized, and in the post-pandemic era) of the COVID-19 pandemic on mental and sexual health is unclear. Therefore, this study aimed to investigate the mental and sexual health of patients with infertility during different stages of the COVID-19 pandemic.

## Materials and methods

### Participants

This prospective before-and-after study was conducted at the Reproductive Medical Center of Shengjing Hospital. The study periods were April and May 2022 (during the lockdown), December and January 2023 (when controls were fully liberalized), and May and August 2023 (in the post-pandemic era). Infertility is defined as the incapacity to conceive after one year of unprotected intercourse [14].

In establishing inclusion criteria, individuals were required to have a confirmed diagnosis of infertility, fall within the age range of 20 to 45 years, be married, and cohabit with their spouses. To mitigate the potential impact of age and endocrine factors on sexual function and health, the study exclusively enrolled participants under the age of 45 who were married and living with their spouses. Conversely, exclusion criteria comprised individuals who withdrew from the study midway or failed to complete the entire questionnaire during each stage, those consuming medications known to affect sexual function and/or mental state (selective serotonin reuptake inhibitors, tricyclic antidepressants, and phosphodiesterase type 5 inhibitors) [15], individuals with clinically diagnosed sexual dysfunction predating their infertility diagnosis, and those with pre-existing psychiatric conditions known to induce sexual dysfunction. All participants were sourced from the pool of patients undergoing assisted reproductive technology treatments at the reproductive medical center, with each participant providing informed consent for their involvement in the study. This study has been approved by the ethics committee (approval number: 2020PS009F) and conforms to the principles of the Declaration of Helsinki.

### Methods

**Fig. 1:**
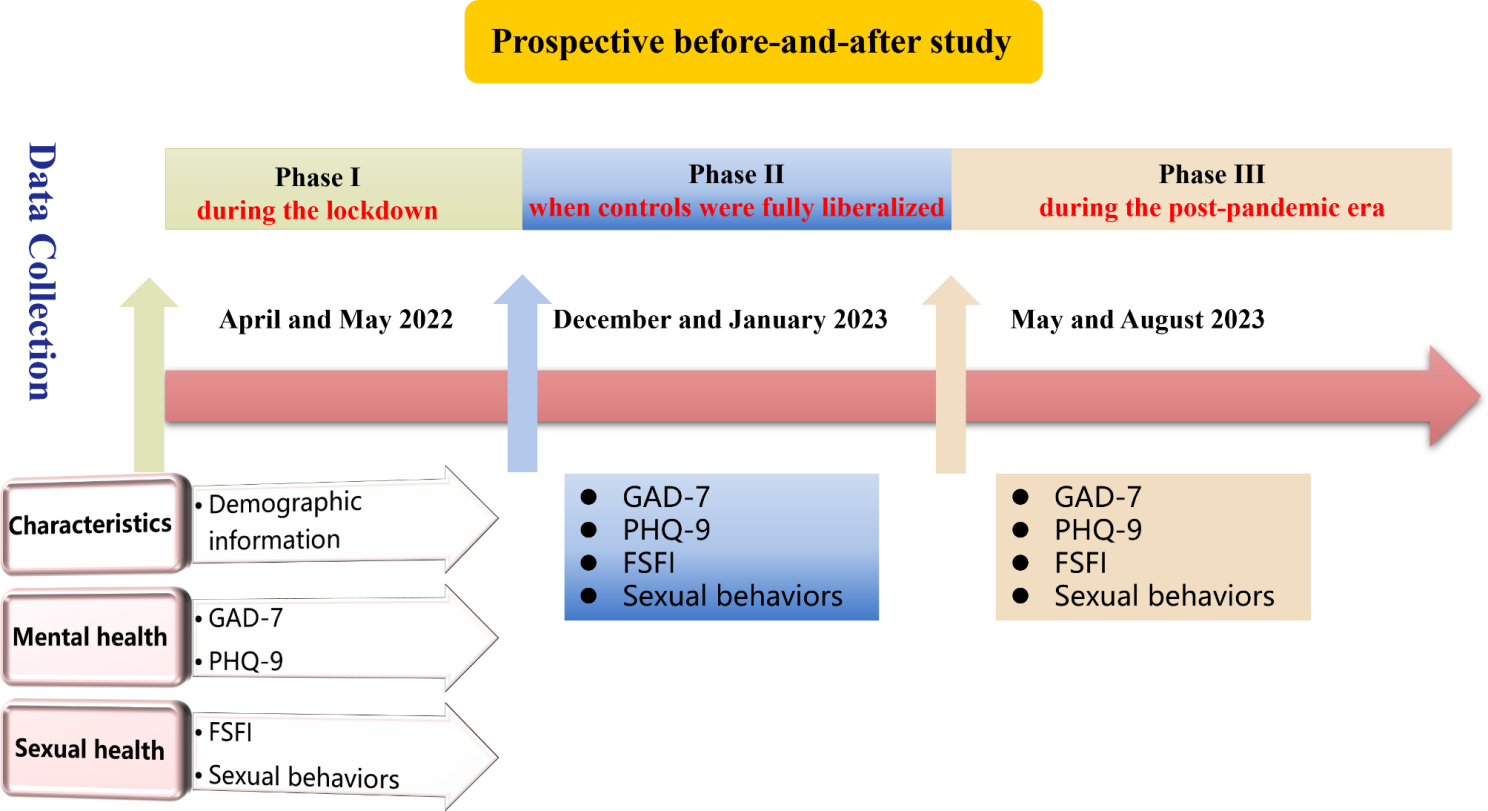
Research flowcharts

This study explored the sexual and mental health of women with infertility during the three stages of the COVID-19 pandemic using questionnaires comprising data of general information about the patients, levels of anxiety and depression, changes in sexual behavior, and levels of sexual function (Fig. 1). Demographic information about the participants included age, height, weight, economic status, duration of infertility, education level, frequency of physical exercise, smoking, and drinking status.

We gauged participants’ levels of anxiety and depression utilizing well-established standardized mental health questionnaires. For the assessment of anxiety symptoms, the 7-item Generalized Anxiety Disorder scale (GAD-7) was employed. Each item was scored on a 4-point scale, ranging from 0 to 3. The cumulative score spanned from 0 to 21, with scores of 5, 10, and 15 signifying mild, moderate, and severe anxiety symptomatology, respectively [16].

To evaluate depression symptoms, we employed the 9-item Patient Health Questionnaire (PHQ-9). Items on the PHQ-9 were scored on a 4-point scale, ranging from 0 to 3. The overall score ranged from 0 to 27, with scores of 5, 10, 15, and 20 denoting mild, moderate, moderate-severe, and severe depression symptom levels, respectively [17]. This systematic approach ensured a comprehensive and nuanced evaluation of participants’ mental health, providing a detailed understanding of both anxiety and depression across various severity levels. The use of established scales and scoring criteria further enhanced the reliability and comparability of the assessments.

To evaluate changes in sexual behavior, we explored participants’ sexual desire, frequency of intercourse, sexual satisfaction, frequency of masturbation, and pornography use across three distinct stages, employing structured questionnaires. Female sexual function was assessed through the Female Sexual Function Index (FSFI), a comprehensive instrument featuring 19 items and six domains (desire, arousal, lubrication, orgasm, satisfaction, and coital pain). This assessment was based on the participants’ sexual experiences in the preceding four weeks [18].

A total FSFI score of ≤ 23.45, aligned with the Chinese cut-off, served as an indicator of potential sexual dysfunction [19, 20]. The Cronbach’s alpha values, surpassing or equal to 0.82, reflected the reliability of the FSFI in measuring female sexual function [18]. This methodology facilitated a thorough examination of various aspects of sexual behavior and function, providing valuable insights into potential changes and challenges experienced by the participants throughout the specified stages.

### Statistical analyses

The data was analyzed using the SPSS statistical software (version 22.0; SPSS Inc., Chicago, IL, USA). Categorical variables were succinctly presented as counts and percentages, while continuous variables were summarized using means and standard deviations (SDs). The Chi-square test was applied to compare categorical data, and the ANOVA was employed for numerical data. To gauge the effect size and ascertain the robustness of each statistical analysis, Cramer’s *V* was calculated for the chi-squared test, and *η²* for the ANOVA. Post-hoc analysis was implemented to assess differences between multiple groups, and a two-tailed *p*-value < 0.05 was considered indicative of statistical significance.

## Results

### Participants’ demographic characteristics

In the first phase, 526 participants participated in the study, of which 352 continued to the second phase. Finally, the third phase had 188 participants because of loss to follow-up or refusal to participate further. Table 1 shows the demographic characteristics of the participants. The average age was 34.68 ± 5.73 years(range from 21 to 45). The average infertility duration was 4.38 ± 3.45 years. (Table 1).

**Table 1:** Demographic characteristics of the study population

Characteristics	n = 188
Age (years)	34.68 ± 5.73
BMI (kg/m^2^)	23.06 ± 2.89
Duration of infertility	4.38 ± 3.45
Primary/ Secondary infertility	
Primary	121 (64.36)
Secondary	67 (35.64)
Region	
Urban	134 (71.28)
Rural	54 (28.72)
Income	
Very low	90 (47.9)
Low	53 (28.2)
Middle	26 (13.8)
High	7 (3.7)
Very high	12 (6.4)
Education	
≤ High school	67 (35.6)
Specialized college	33 (17.6)
College	69 (36.7)
≥ Postgraduate	19 (10.1)
Pressure	
Very high	10 (5.3)
High	42 (22.3)
General	93 (49.5)
Low	29 (15.4)
None	14 (7.4)
Physical exercise frequency	
None	62 (33.0)
2 times a month or less	56 (29.8)
1 time a week	30 (16.0)
2 times a week and above	40 (21.3)
Smoking status	
Smoker	6 (3.2)
Non-smoker	182 (96.8)
Drinking alcohol	
Often	0
Sometimes	21 (11.2)
Rarely	68 (36.2)
Never	99 (52.7)

Data was described as mean ± SD or n (%)

Abbreviations: SD: standard deviation; BMI: body mass index

### The level of mental health of the participants in the three phases

The GAD-7 scores were 6.77 ± 3.57 in the first stage, 11.82 ± 3.56 in the second stage, and 6.15 ± 2.87 in the third stage. In the first stage, the incidence rate of moderate-to-severe anxiety was 11.3%; second stage, 68.6%; and third stage, 11.1%. The incidence of anxiety was highest in the second phase (*p* < 0.01). The PHQ-9 score was 8.32 ± 4.30 in the first stage, 14.20 ± 4.37 in the second stage, and 8.77 ± 4.31 third stage. The incidence rate of moderate-to-severe depression was 9.1% in the first stage, 45.8% in the second stage, and 7.5% in the third stage. The incidence of depression was highest in the second phase (*p* < 0.01). (Table 2).

**Table 2:** The level of mental health of the participants in the three phases

Items	Phase I n (%)	Phase II n (%)	Phase III n (%)	Statistics	Effect size (*η2* / *V*)	*P*-value
GAD-7 score	6.77 ± 3.57	11.82 ± 3.56 ^a^	6.15 ± 2.87 ^c^	*F* = 162.08	*η²* = 0.37	< 0.01^**^
Level of anxiety				*χ*^*2*^ = 213.33	*V* = 0.44	< 0.01^**^
None	39 (20.7)	1 (0.5)	56 (29.8)			
Mild	126 (67.0)	58 (30.9)	111 (59.0)			
Moderate	8 (4.3)	87 (46.3)	17 (9.0)			
Severe	15 (8.0)	42 (22.3)	4 (2.1)			
PHQ-9 score	8.32 ± 4.30	14.20 ± 4.37 ^a^	8.77 ± 4.31 ^c^	*F* = 107.56	*η²* = 0.28	< 0.01^**^
Level of depression				χ^2^ = 193.71	*V* = 0.52	< 0.01^**^
None	16 (8.5)	2 (1.1)	21 (11.2)			
Mild	123 (65.4)	23 (12.2)	103 (54.8)			
Moderate	32 (17.0)	77 (41.0)	50 (26.6)			
Moderately-severe	9 (4.8)	65 (34.6)	6 (3.2)			
Severe	8 (4.3)	21 (11.2)	8 (4.3)			

^**^p < 0.01

GAD-7: 7-item Generalized Anxiety Disorder Scale

PHQ-9: 9-item Patient Health Questionnaire

a: Phase I compared with Phase II had a significant difference

c: Phase II compared with Phase III had a significant difference

### Changes in sexual behavior of patients with infertility during different stages

In the three stages, subjects reported decreases in sexual desire of 12.2%, 27.1%, and 12.2%, respectively (*p* < 0.01). Regarding the decrease in sexual frequency, the incidence rates were 12.8%, 27.1%, and 11.7% in the three stages, respectively (*p* < 0.01). For a decrease in sexual satisfaction, the incidence was 9.6%, 18.1%, and 9.0% in the three stages, respectively (*p* < 0.01). (Table 3).

**Table 3:** Changes in sexual behavior of patients with infertility during different stages

Items	Phase I n (%)	Phase II n (%)	Phase III n (%)	Statistics	Effect size (*V*)	*P*-value
Changes in sexual desire				*χ*^*2*^ = 31.94	*V* = 0.17	< 0.01^**^
Decreased	23 (12.2)	51 (27.1)	23 (12.2)			
Unchanged	150 (79.8)	137 (72.9)	150 (79.8)			
Increased	15 (8.0)	0	18 (8.0)			
Changes in sexual frequency				*χ*^*2*^ = 32.50	*V* = 0.17	< 0.01^**^
Decreased	24 (12.8)	51 (27.1)	22 (11.7)			
Unchanged	149 (79.3)	137 (72.9)	150 (79.8)			
Increased	15 (8.0)	0	16 (8.5)			
Changes in sexual satisfaction				*χ*^*2*^ = 19.60	*V* = 0.13	< 0.01^**^
Decreased	18 (9.6)	34 (18.1)	17 (9.0)			
Unchanged	158 (84.0)	154 (81.9)	160 (85.1)			
Increased	12 (6.4)	0	11 (5.9)			
Changes in frequency of masturbation				*χ*^*2*^ = 8.81	*V* = 0.09	0.19
Increased	37 (19.7)	35 (18.6)	23 (12.2)			
Unchanged	63 (33.5)	65 (34.6)	69 (36.7)			
Decreased	11 (5.9)	22 (11.7)	20 (10.6)			
None	77 (41.0)	66 (35.1)	76 (40.4)			
Changes in frequency of pornography use				*χ*^*2*^ = 7.99	*V* = 0.08	0.24
Increased	27 (14.4)	31 (16.5)	19 (10.1)			
Unchanged	42 (22.3)	40 (21.3)	50 (26.6)			
Decreased	13 (6.9)	23 (12.2)	16 (8.5)			
None	106 (56.4)	94 (50.0)	103 (54.8)			

^**^p < 0.01

### Sexual function of patients with infertility during different stages

Regarding female sexual health, the total FSFI scores of the subjects in the three stages were 27.02 ± 4.19, 27.29 ± 4.00, and 24.33 ± 4.37, respectively. The scores for the six domains of female sexual function (sexual desire, sexual arousal ability, vaginal lubrication, orgasm, satisfaction, and coital pain) varied significantly among the three groups, with these being lowest in the second stage (*p* < 0.01). As a result, the incidence of sexual dysfunction in the second stage (34.6%) was significantly higher than that in the first (18.6%) and third stages (16.0%) (*p* < 0.01) (Table 4).

**Table 4:** Sexual function of patients with infertility during different stages

Items	Phase I n (%)	Phase II n (%)	Phase III n (%)	Statistics	Effect size (*η2* / *V*)	*P*-value
FSFI score	27.02 ± 4.19	24.33 ± 4.37^a^	27.29 ± 4.00 ^c^	*F* = 28.70	*η²* = 0.09	< 0.01^**^
Sexual desire score	3.46 ± 0.76	2.98 ± 0.85 ^a^	3.84 ± 0.90 ^b,c^	*F* = 49.75	*η²* = 0.15	< 0.01^**^
Sexual arousal ability score	4.09 ± 0.96	3.59 ± 0.99 ^a^	4.14 ± 1.02 ^c^	*F* = 17.80	*η²* = 0.06	< 0.01^**^
Vaginal lubricity score	5.18 ± 0.81	4.86 ± 0.98 ^a^	5.17 ± 0.77 ^c^	*F* = 8.57	*η²* = 0.03	< 0.01^**^
Orgasm score	4.62 ± 0.99	4.16 ± 1.03 ^a^	4.60 ± 0.98 ^c^	*F* = 12.70	*η²* = 0.04	< 0.01^**^
Sexual satisfaction score	4.66 ± 1.04	4.01 ± 1.06 ^a^	4.62 ± 1.05 ^c^	*F* = 22.72	*η²* = 0.08	< 0.01^**^
Coital pain score	5.02 ± 0.96	4.74 ± 1.05 ^a^	4.93 ± 0.99 ^c^	*F* = 3.86	*η²* = 0.01	< 0.05^*^
Incidence of sexual dysfunction	35 (18.6)	65 (34.6)	30 (16.0)	*χ*^*2*^ = 0.16	*V* = 0.20	< 0.01^**^

^*^p < 0.05 ^**^p < 0.01

a: Phase I compared with Phase II had a significant difference

b: Phase I compared with Phase III had a significant difference

c: Phase II compared with Phase III had a significant difference

## Discussion

In this prospective before-and-after study, we assessed the mental and sexual health of individuals with infertility during different stages of the COVID-19 pandemic. Using standardized mental health and sexual function questionnaires, we found that participants had the highest rates of anxiety, depression, and sexual dysfunction when the COVID-19 control measures were fully liberalized. Our study is the first to report on the impact of COVID-19 on the mental and sexual health of patients with infertility during different stages of the pandemic.

Patients with infertility form a particular group in society, and the diagnosis of infertility can be stressful and can impair mental and sexual health [21]. Thus, the impact of COVID-19 on the mental and sexual health of such patients is a complex and sensitive topic. During the COVID-19 pandemic, these issues are likely to be further exacerbated by factors such as health concerns, social isolation, and life uncertainty [13, 22]. Previously, we explored the impact of the COVID-19 pandemic on the sexual and mental health of people with infertility through cross-sectional studies and found that postponing fertility treatment due to the COVID-19 pandemic had a significant impact on sexual life, mental health, and couple relationships [23]. Additionally, the COVID-19 pandemic had a significant impact on the sexual behavior of people with infertility [24].

Mental and sexual health is affected by many external factors, such as stressful events, couple and family relationships, financial level, etc. [24]. It is difficult to draw accurate conclusions on the impact of stressful events (such as the COVID-19 pandemic) through cross-sectional studies of populations, and before-and-after studies can better explore the impact of changes in stressful events on specific populations. By comparing the mental and sexual health levels of the same population during the three phases of the event (during the pandemic, full liberalization, and the post-pandemic era), we can more accurately determine the trend in the health level of patients during this period.

When control measures were fully released, the declines in the frequency of sexual intercourse and desire were significantly higher than that during the pandemic and the post-pandemic era. This may be because, when the lockdown was just lifted, a large population may have been infected, and the number of infected patients may have increased sharply, making it difficult to seek medical treatment, buy medicines, etc. This might have led to severe psychological pressure, which in turn caused changes in sexual behavior.

Owing to anxiety, stress, and life changes, people with infertility may experience sexual health problems, including decreased libido, sexual dysfunction, or decreased sexual satisfaction. Previous studies have explored the impact of the COVID-19 pandemic on changes in sexual behavior in different social groups. Studies have reported a significant decrease in sex frequency during the COVID-19 pandemic compared with that during the pre-COVID-19 period [25, 26]. However, changes in sexual frequency and desire were most significant when control was fully liberalized.

In our study, the highest rates of anxiety and depression were found in patients with infertility when the COVID-19 control measures were fully liberalized. The COVID-19 pandemic has impaired the mental health of the population, with a significant increase in the prevalence and burden of major depression and anxiety. Most people experienced mild-to-severe anxiety and depression throughout the pandemic phase. When control was fully lifted, almost 99% of participants had mild-to-severe anxiety and depression. This symptom plateaued in the post-pandemic era with no significant difference compared to that in the lockdown period. Therefore, it is likely that the cause of anxiety and depression at these two stages may be infertility itself.

With complete liberalization of controls, infections can break out in full swing, and patients with infertility may experience inadequate medical resources, which may affect their treatment plans and ability to obtain professional help. In such emergencies, patients may not have access to their usual emotional support, which is important for managing infertility and coping with the psychological stress of the pandemic.

These findings hold significant relevance for individuals experiencing infertility, particularly during challenging times. Preserving mental and sexual well-being is crucial for those facing infertility. It underscores clinicians’ importance in prioritizing mental health support and offering interventions such as psychotherapy and counseling. This proactive approach aims to assist individuals in coping with anxiety, stress, and emotional distress, recognizing the intricate interplay between mental and sexual health in the context of infertility. This study underscores the importance of early screening and psychosocial intervention within the realm of infertility. By identifying and addressing potential risk factors early on, we aim to prevent the development of mental health issues and sexual dysfunction. The hope is that our findings contribute to a heightened awareness of the intricate connections between infertility, mental well-being, and sexual health, ultimately paving the way for more effective and timely interventions to support individuals facing these challenges.

Individuals experiencing infertility are encouraged to prioritize open communication with their partners, sharing emotions and needs openly. Establishing a solid support system is vital, fostering an environment where mutual understanding and support prevail. Adopting and sustaining healthy lifestyle habits, such as maintaining a nutritious diet, ensuring adequate sleep, and engaging in moderate exercise, plays a crucial role in both physical and mental well-being. Despite the challenges posed by infertility, cultivating a positive mindset and maintaining hope for the future are pivotal for mental health. Consideration of activities like emotional therapy and meditation can contribute significantly to emotional well-being. By embracing these practices, individuals navigating infertility can enhance their overall resilience and foster a more positive outlook on their journey.

## Conclusion

This is the first study to report the repercussions of COVID-19 on the mental and sexual well-being of individuals experiencing infertility across various phases of the pandemic. Upon the complete lifting of control measures, close to 99% of participants exhibited varying degrees of anxiety and depression. Our research underscores that individuals with infertility faced elevated levels of anxiety, depression, and sexual dysfunction during the phase of full liberalization of COVID-19 control measures, in stark contrast to the periods of lockdown and the post-pandemic era.

### Electronic supplementary material

Below is the link to the electronic supplementary material.

Supplementary Material 1

## Data Availability

The datasets used and/or analyzed during the current study are available from the first author on reasonable request.
