# Supplementary material for: The impact of COVID-19 on the mental and sexual health of patients with infertility: a prospective before-and-after study
Source: Reprod Biol Endocrinol. 2024 Jan 2;22:1. doi: 10.1186/s12958-023-01174-7 (PMC10759678; doi:10.1186/s12958-023-01174-7)
Supplement: Supplementary file 1 — Supplementary Material 1 [file 12958_2023_1174_MOESM1_ESM.doc]

**Sexual Health and Psychological Health Survey Questionnaire**

**(for female)**

We are very grateful for your contributions to this research, and wish you all the best and realize your dreams soon.

Age: Height: cm Weight: kg

What type of profession do you do?

□Civil servant □Professional and technical personnel □Business management personnel □Staff □Worker □Farmer □Self-employed □None □Others: ____________

Personal annual income □<50,000 yuan □50,000-100,000 yuan □100,000-150,000 yuan □150,000-200,000 yuan □>200,000 yuan

How many years have you planed to have a child?

: _________ years Entertainment: ___________

Do you take drugs? □Yes, what kind of drugs:___________ □No

Time spent on cell phones per day: ___________ hours

Sexual life frequency: ___________ times per month

Education level □high school and below □college for professional training □undergraduate □postgraduate and above

**For the following questions, please choose a check mark before the choice that you think best suits your situation during COVID-19 pandemic.**

Diet □ only vegetarian food □ mainly meat □ half vegetarian food and half vegetarian meat

Is there a big stress in work and life? □very high □high □general □low □no

Frequency of physical exercise □None □2 times a month or less

□1 time a week □2 times a week and above

Smoking □Yes,______ cigarettes per day □No

Drinking □Almost every day □ Often □ Sometimes □ Rarely □ Never

Coffee □Almost every day □Always □Sometimes □Rarely □Never

1. Does the novel coronavirus pneumonia pandemic make you anxious?

□Severe □Slight □None

2. Compared with that before the COVID-19 pandemic, how is your relationship with your partner?

□Very good □Fair □Deteriorating

3. During the novel coronavirus pneumonia pandemic, how does your relationship compared to before?

□Better □No change □Worse

4. Compared with that before the COVID-19 pandemic, how has your sexual desire changed?

□Increased □Unchanged □Decreased

5. Compared with that before the COVID-19 pandemic, how has your sexual frequency changed?

□Increased □Unchanged □Decreased

6. Compared with that before the COVID-19 pandemic, how has your sexual satisfaction changed?

□Increased □Unchanged □Decreased

7. During the new coronavirus pneumonia pandemic, what about your drinking before or during sexual activity?

□Increased □Unchanged □Decreased

8. How has your frequency of masturbation changed compared with that before the COVID-19 pandemic?

□Increased □Unchanged □Decreased □ None

9. How has your frequency of pornography use changed compared with that before the COVID-19 pandemic?

□Increased □Unchanged □Decreased □ None

10. During the new coronavirus pneumonia pandemic, how does your condom use frequency (in sexual contact) change?

□Increased □Unchanged □Decreased

11.How is your annual income affected by the new coronavirus pneumonia?

□Increased □Unchanged □Decreased

12. Have you postponed your plan to have a child because of the new coronary pneumonia? How long？

□Yes □No

13. Have you encountered difficulties in receiving fertility treatment due to new coronary pneumonia?

□Yes □No

If you have, what are the difficulties? ________________________________________________

14. In the process of receiving fertility treatment, has the new coronavirus nucleic acid test increased your burden?

□Yes □No

15. Is sex important to you?

□Very important □Important □General □Unimportant □Very unimportant

16. Over the past 4 weeks, how often did you feel sexual desire or interest?

□Almost always or always

□Most times (more than half the time)

□Sometimes (about half the time)

□A few times (less than half the time)

□Almost never or never

17. Over the past 4 weeks, how would you rate your level (degree) of sexual desire or

interest?

□Very high □High □Moderate □Low □Very low or none at all

18.Over the past 4 weeks, how often did you feel sexually aroused (“turned on”) during sexual activity or intercourse?

□Almost always or always

□Most times (more than half the time)

□Sometimes (about half the time)

□A few times (less than half the time)

□Almost never or never

19.Over the past 4 weeks, how would you rate your level of sexual arousal (“turn on”)

during sexual activity or intercourse?

□No sexual activity

□Very high

□High

□Moderate

□Low

□Very low or none at all

20.Over the past 4 weeks, how confident were you about becoming sexually aroused

during sexual activity or intercourse?

□ No sexual activity

□ Very high confidence

□ High confidence

□ Moderate confidence

□ Low confidence

□ Very low or no confidence

21.Over the past 4 weeks, how often have you been satisfied with your arousal (excitement) during sexual activity or intercourse? Response Options

□ No sexual activity

□Almost always or always

□Most times (more than half the time)

□Sometimes (about half the time)

□A few times (less than half the time)

□Almost never or never

22.Over the past 4 weeks, how often did you become lubricated (“wet”) during sexual

activity or intercourse?

□ No sexual activity

□Almost always or always

□Most times (more than half the time)

□Sometimes (about half the time)

□A few times (less than half the time)

□Almost never or never

23.Over the past 4 weeks, how difficult was it to become lubricated (“wet”) during sexual activity or intercourse?

□ No sexual activity

□ Extremely difficult or impossible

□ Very difficult

□ Difficult

□ Slightly difficult

□ Not difficult

24.Over the past 4 weeks, how often did you maintain your lubrication (“wetness”) until

completion of sexual activity or intercourse?

□ No sexual activity

□Almost always or always

□Most times (more than half the time)

□Sometimes (about half the time)

□A few times (less than half the time)

□Almost never or never

25.Over the past 4 weeks, how difficult was it to maintain your lubrication (“wetness”)

until completion of sexual activity or intercourse?

□ No sexual activity

□ Extremely difficult or impossible

□ Very difficult

□ Difficult

□ Slightly difficult

□ Not difficult

26.Over the past 4 weeks, when you had sexual stimulation or intercourse, how often

did you reach orgasm (climax)?

□ No sexual activity

□Almost always or always

□Most times (more than half the time)

□Sometimes (about half the time)

□A few times (less than half the time)

□Almost never or never

27.Over the past 4 weeks, when you had sexual stimulation or intercourse, how diffi-

cult was it for you to reach orgasm (climax)?

□ No sexual activity

□ Extremely difficult or impossible

□ Very difficult

□ Difficult

□ Slightly difficult

□ Not difficult

28.How satisfied have you been with the amount of emotional closeness during sexual activity between you and your partner?

□ No sexual activity

□ Very satisfied

□ Moderately satisfied

□About equally satisfied and dissatisfied

□ Moderately dissatisfied

□ Very dissatisfied

29.Over the past 4 weeks, how satisfied have you been with the amount of emotional closeness during sexual activity between you and your partner?

□ No sexual activity

□ Very satisfied

□ Moderately satisfied

□About equally satisfied and dissatisfied

□ Moderately dissatisfied

□ Very dissatisfied

30.how satisfied have you been with your sexual relationship with your partner?

□ Very satisfied

□ Moderately satisfied

□About equally satisfied and dissatisfied

□ Moderately dissatisfied

□ Very dissatisfied

31.Over the past 4 weeks, how satisfied have you been with your overall sexual life?

□ Very satisfied

□ Moderately satisfied

□About equally satisfied and dissatisfied

□ Moderately dissatisfied

□ Very dissatisfied

32.Over the past 4 weeks, how often did you experience discomfort or pain during

vaginal penetration?

□ Did not attempt intercourse

□ Almost always or always

□ Most times (more than half the time)

□ Sometimes (about half the time)

□ A few times (less than half the time)

□ Almost never or never

33.Over the past 4 weeks, how often did you experience discomfort or pain following vaginal penetration?

□ Did not attempt intercourse

□ Almost always or always

□Most times (more than half the time)

□ Sometimes (about half the time)

□ A few times (less than half the time)

□ Almost never or never

34.Over the past 4 weeks, how would you rate your level (degree) of discomfort or pain

during or following vaginal penetration?

□ Did not attempt intercourse

□ Very high

□ High

□ Moderate

□ Low

□Very low or none at all

35. Intercourse time

□<1 minute □1-2 minutes □3-4 minutes □5-7 minutes □8-10 minutes

□11-15 minutes □16-30 minutes □>30 minutes

36. Foreplay time

□<1 minute □2-10 minutes □11-20 minutes □21-30 minutes □31-60 minutes

□>60 minutes

37. The possibility of reaching orgasm through masturbating.

□Almost always □Always □Sometimes □Rarely □Never □Never tried

38. The possibility of reaching orgasm in non-coital intercourse (such as foreplay or oral sex)

□Almost always □Always □Sometimes □Rarely □Never □Never tried

39. The possibility of having an orgasm during sexual intercourse

□Almost always □Always □Sometimes □Rarely □Never □Never tried

**The Generalized Anxiety Disorder Screener (GAD-7)**

In the past two weeks, how often did the following symptoms appear in your life?

1.Feeling nervous, anxious or on edge

□No □A few days □More than half of the time □Almost every day

2.Not being able to stop or control worrying

□No □A few days □More than half of the time □Almost every day

3.Worrying too much about different things

□No □A few days □More than half of the time □Almost every day

4.Trouble relaxing

□No □A few days □More than half of the time □Almost every day

5.Being so restless that it is hard to sit still

□No □A few days □More than half of the time □Almost every day

6.Becoming easily annoyed or irritable

□No □A few days □More than half of the time □Almost every day

7.Feeling afraid as if something awful might happen

□No □A few days □More than half of the time □Almost every day

**The Patient Health Questionnaire-9(PHQ-9)**

In the past two weeks, how often did the following symptoms appear in your life?

1 Little interest or pleasure in doing things

□No □A few days □More than half of the time □Almost every day

2 Feeling down, depressed, or hopeless

□No □A few days □More than half of the time □Almost every day

3 Difficulty falling or staying asleep, or sleeping too much

□No □A few days □More than half of the time □Almost every day

4 Feeling tired or having little energy

□No □A few days □More than half of the time □Almost every day

5 Poor appetite or overeating

□No □A few days □More than half of the time □Almost every day

6 I am dissatisfied with myself, feel that I am a failure, or embarrass my family

□No □A few days □More than half of the time □Almost every day

7 Trouble concentrating on things, such as reading the newspaper or watching TV

□No □A few days □More than half of the time □Almost every day

8 Moving or speaking so slowly that other people could have noticed? Or the opposite—being so fidgety or restless that you have been moving around a lot more than usual.

□No □A few days □More than half of the time □Almost every day

9 Thoughts that you would be better off dead or of hurting yourself in some way

□No □A few days □More than half of the time □Almost every day
